# Cardiovascular prevention model from Kenyan slums to migrants in the Netherlands

**DOI:** 10.1186/s12992-015-0095-y

**Published:** 2015-03-07

**Authors:** Steven van de Vijver, Samuel Oti, Eric Moll van Charante, Steven Allender, Charlie Foster, Joep Lange, Brian Oldenburg, Catherine Kyobutungi, Charles Agyemang

**Affiliations:** African Population and Health Research Center, Nairobi, Kenya; Department of Global Health, Academic Medical Center, Amsterdam Institute for Global Health and Development, University of Amsterdam, Amsterdam, The Netherlands; Department of Family Medicine, Academic Medical Center, University of Amsterdam, Amsterdam, The Netherlands; Department of Public Health, Deakin University, Melbourne, Australia; Nuffield Department of Population Health, University of Oxford, Oxford, United Kingdom; Melbourne School of Population and Global Health, University of Melbourne, Melbourne, Australia; Department of Public Health, Academic Medical Center, University of Amsterdam, Amsterdam, The Netherlands

**Keywords:** Reverse innovation, Hypertension, Prevention, Treatment, Slums, Kenya, African migrants

## Abstract

Cardiovascular diseases (CVD) are the main cause of morbidity and mortality worldwide. As prevention and treatment of CVD often requires active screening and lifelong follow up it is a challenge for health systems both in high-income and low and middle-income countries to deliver adequate care to those in need, with efficient use of resources.

We developed a health service model for primary prevention of CVD suitable for implementation in the Nairobi slums, based on best practices from public health and the private sectors. The model consists of four key intervention elements focusing on increasing awareness, incentives for promoting access to screening and treatment, and improvement of long-term adherence to prescribed medications. More than 5,000 slum dwellers aged ≥35 years and above have been screened in the study resulting in more than 1000 diagnosed with hypertension and referred to the clinic.

Some marginalized groups in high-income countries like African migrants in the Netherlands also have low rates of awareness, treatment and control of hypertension as the slum population in Nairobi. The parallel between both groups is that they have a combination of risky lifestyle, are prone to chronic diseases such as hypertension, have limited knowledge about hypertension and its complications, and a tendency to stay away from clinics partly due to cultural beliefs in alternative forms of treatment, and lack of trust in health providers. Based on these similarities it was suggested by several policymakers that the model from Nairobi can be applied to other vulnerable populations such as African migrants in high-income countries. The model can be contextualized to the local situation by adapting the key steps of the model to the local settings.

The involvement and support of African communities’ infrastructures and health care staff is crucial, and the most important enabler for successful implementation of the model in migrant communities in high-income countries. Once these stakeholders have expressed their interest, the impact of the adapted intervention can be measured through an implementation research approach including collection of costs from health care providers’ perspective and health effects in the target population, similar to the study design for Nairobi.

**Electronic supplementary material:**

The online version of this article (doi:10.1186/s12992-015-0095-y) contains supplementary material, which is available to authorized users.

## Background

Cardiovascular diseases (CVD) are the main causes of morbidity and mortality worldwide [1]. Although they have been seen traditionally as diseases of high income countries, there has been an increasing shift in the burden of CVD to low and middle income countries (LMICs) [2]. This has led to a dramatic increase in the number of people at risk for developing CVD across the globe. As prevention and treatment of CVD often requires active screening and lifelong follow up, this is a challenge to the health services both of high-income and LMICs to deliver adequate care to those in need, with efficient use of resources. Our paper describes the development of a novel community based CVD prevention program in slums of Kenya and how this approach could be adapted to vulnerable migrant populations in high-income countries such as in the Netherlands.

### Development of SCALE UP project in slums of Nairobi

Urbanization is recognized as an important cause for CVD in LMIC. In Africa, being the fastest urbanizing continent, lifestyle regarding diet and physical exercise is changing rapidly, leading to increased rates of CVD and its risk factors such as hypertension, obesity and diabetes [3-5]. Evidence indicates that more than 60% of the urban population in many African countries lives in slums [6], which are characterized by high population density, overcrowding, poverty, high mobility, insecurity, social exclusion, and poor infrastructure such as lack of safe water and sanitation systems. Due to these poor circumstances, infectious diseases including tuberculosis, pneumonia, hepatitis and diarrheal disease are still rampant in the slums. In addition, non-communicable diseases such as CVD and diabetes are also becoming much more common in such settings, hence creating a double burden of disease. This is further aggravated by interactions between infectious diseases and NCDs like for example TB with diabetes.

**Figure 1:**
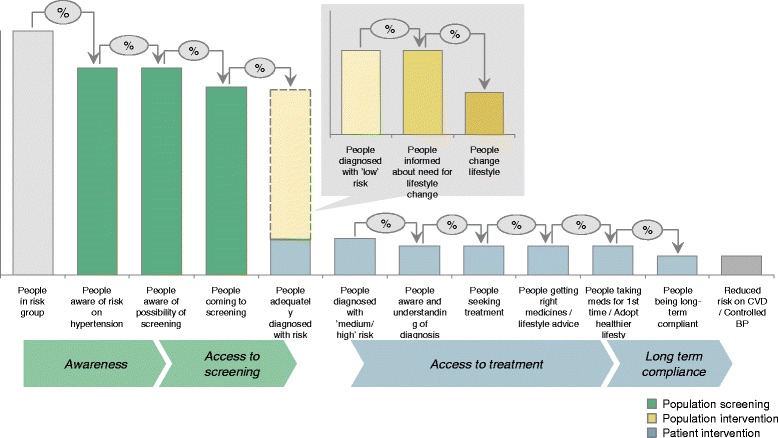
**Framework showing steps from awareness of cardiovascular risk factors in risk group towards long term compliance with controlled blood pressure.**

In spite of the tremendous need, access to healthcare and social services are generally lacking in these settings. To address some of these health challenges in slum communities in Africa, two public health research organizations, the African Population and Health Research Center (APHRC) and the Amsterdam Institute for Global Health and Development (AIGHD) have collaborated on a joint program. Between 2011 and 2012, they have developed a health service package for primary prevention of CVD suitable for implementation in the Nairobi slums, in collaboration with a private sector partner, the Boston Consulting Group (BCG) [7]. The aim of this “hybrid” collaboration was to integrate public and private sector approaches in order to develop an innovative health service delivery package for CVD prevention for the urban poor on the African continent. As resources are limited in these settings it was essential that the final model should be affordable, feasible, and cost-effective. A conceptual framework was developed based on previous studies on CVD risk factors in this setting [8-10], a comprehensive literature review on the effectiveness of community based CVD prevention programs in LMICs [11], and the local experiences of an intervention project to improve patient access to treatment for hypertension and diabetes in primary care settings in the slums of Nairobi (Figure 1).

This framework provides an overview of all the different steps from awareness of cardiovascular risk factors like hypertension through, to treatment adherence and successful blood pressure control. Following a literature review and consultation with local stakeholders we identified the main bottlenecks contributing to low health service utilization. Four key issues were identified: 1) increasing awareness of CVD risk, 2) improving access to screening, 3) facilitating access to treatment, and 4) adhering to prescribed medication.

**Figure 2:**
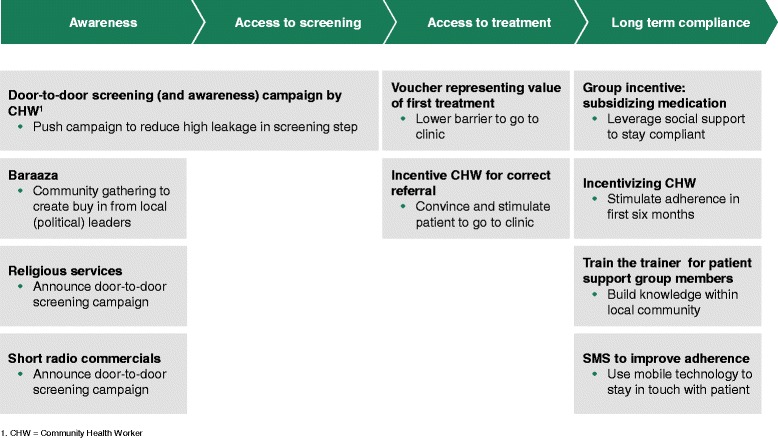
**Overview of interventions in the final SCALE UP model.**

We organized sessions with all the relevant stakeholders on CVD prevention including the representatives of the local community, patient groups, health staff and community health workers, academic experts and program implementers from the public and private sector and policymakers from the Ministry of Health. Based on the input from these stakeholders, we identified the most affordable, feasible and cost-effective health service delivery package for prevention of CVD in the slums of Nairobi. The final model involved a multi-component intervention with four key elements focusing on increasing awareness, incentives for access to screening and treatment, and improvement of long term adherence to prescribed medications (Figure 2).

During the development and subsequent implementation of the new model in the slums of Nairobi, we encountered several challenges, not least because of the dynamic nature of the environment (Van de Vijver et al., unpublished). Due to the extreme poverty in the slums there are interrelated issues like insecurity, high mobility of the population, lack of infrastructure, multi-morbidity and local politics that have the potential to jeopardize the project.

**Figure 3:**
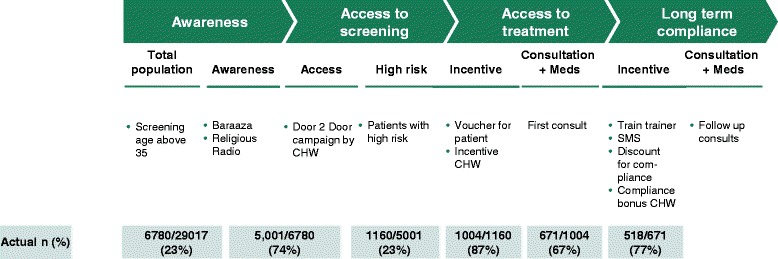
**Actual percentages and numbers in the intervention.**

The preliminary analyses of the study suggest promising results on the four identified issues. Awareness creation in the slum has resulted in relatively high percentages of follow up. For instance, 74% of the eligible population was screened of which 23% had hypertension. In 87% of this group the hypertension was confirmed during the second visit. These confirmed hypertensive patients were given free vouchers for the clinic. From the 1004 vouchers that were given to the patients, 67% of the patients showed up for a first time visit, and 77% of these 671 patients showed up on follow up visits (Figure 3). Overall this has resulted in improved quality of care for hypertension patients in the slums of Nairobi.

### Applicability of the SCALE UP model for use with African migrants in other contexts and settings

As our initial aim was to develop a model that could be easily scaled up to other settings in the region, the team also developed a manual (see Additional file 1) that could be used to adapt the approach to other resource poor settings according to the characteristics and features of the local context. It was during meetings with relevant international policymakers that it was suggested that this model might also be applicable to disadvantaged populations in middle or even in high-income countries. From the discussion it became clear that some marginalized groups in high-income countries (e.g. migrant population in Europe) share similar characteristics with the slum populations despite living in different contexts in terms of migration experience, poor socio-economic circumstances, social exclusion, lack of trust and poor access to health and social services [12,13] despite the apparently differences in the levels of poverty. They also have similar low rates of control of hypertension [14,15] and are not benefiting equally from the existing health systems. The potential of this so called reverse innovation of implementing health programs from low income countries to high income countries is supported by a growing body of evidence [16].

Thus, we believe that the SCALE UP model might also be suitable for vulnerable populations such as migrants in high income countries. The current existing health structure in migrant communities such as in Amsterdam Zuid-Oost in the Netherlands where a substantial number of African migrants live provides treatment and care for hypertension in well-equipped primary care clinics, but there is currently no community-based approach to actively raise awareness and do screenings in the population at risk, which would make the case for an introduction of SCALE UP. The parallel between the slum population in Nairobi and the migrant community in Amsterdam is that both of them have a combination of risky lifestyle, are prone to chronic diseases such as hypertension, have limited knowledge about hypertension and its complications, and a high tendency to stay away from clinics partly due to cultural beliefs in alternative treatment and lack of trust in health providers [17].

The SCALE UP model can therefore be applied among vulnerable African migrant populations in high-income countries by following the different steps of the manual in order to adapt the content to the local situation. First, the key risk factors for CVD should be determined, which, based on the existing literature, seems to be hypertension [14,15]. The second step will be to tailor the awareness campaign to the local situation, with potential entry points being the churches, local television and radio, community leaders and cultural gatherings. Consequently the cost-effectiveness of the different options for awareness campaign should be determined. The third step will be tailoring the access to screening method, which might involve screening points in churches and community centers. Part of the development of the screening method will also be determining the target age group. The fourth step is to adapt the stimulus of access to treatment to the situation of African migrants, which could include group consultations, financial and non-financial incentives and follow up calls for high risk patients. The fifth step in the adjusted SCALE UP model will be to tailor the incentives for long term compliance, which could also include SMS reminders and development of patient support groups.

Finally the cost-effectiveness of the tailored SCALE UP model for the African migrants should be determined and the content discussed with the local community and policymakers, as it is essential that this kind of approach is supported by the target population.

As there is a significant difference in the health budget of Nairobi and the Netherlands, respectively just 5% of their budget versus 15% of their GDP, this might have a clear impact as well on different elements in the intervention for example leading to an increased access to treatment in The Netherlands.

The involvement and support of African communities’ infrastructures and local policymakers and health care staff is crucial, and the most important enabler, for successful implementation of the SCALE UP model in migrant communities in high-income countries like the Netherlands [18]. In order to get the support from these actors it is essential that they clearly see the potential health benefits and financial savings of this approach, and take ownership in the development and the implementation of the model. In this process they will be facilitated by experienced academics and implementers, ideally from partner organizations that developed the original model.

### Potential impact/benefits

The potential applicability of the SCALE UP model to vulnerable migrant populations in high income countries was recognized, despite differences in context and specific disease profiles like the infectious burden, reflecting the similarities in social circumstances and health outcomes on cardiovascular risk among slum dwellers in LMICs and migrant communities in high-income countries. In addition, similar challenges were identified among both groups in cultural beliefs and perceptions on hypertension and prevention, and passive attitudes towards the existing health care facilities. The potential impact of the SCALE UP model is that it might successfully address this resemblance by increasing the uptake of screening, treatment and control of hypertension among African migrants. As this innovative approach proves to be effective to include this marginalized group into the local health system it might potentially be scaled up to other relevant health topics like screening and treatment of sexually transmitted diseases (STI’s) and depression and related mental health issues.

The innovation fits well within the health system context among African migrants in high-income countries as there is a tendency to decentralize health care and increase patient participation and ownership, for example by installing blood pressure screening at churches or community centers and organize patient support groups, in order to increase compliance to medication and healthy lifestyle behavior. The impact of the SCALE UP model among African migrants can be conceptualized and measured in the following steps. Once the African migrant community, the local policymakers and health care practitioners have expressed their interest, the SCALE UP model can be tailored to the local situation based on cost-effectiveness calculations. The impact of the adapted intervention will be measured through an implementation research approach including collection of costs from health care providers’ perspective and health effects in the target population, similar to the study design for Nairobi [19].

## Conclusion

With growing urbanization and globalization, the biggest burden of CVD will be among the urban poor in both high-income and LMICs. A community based model for prevention of CVD for the slums in Nairobi was developed, based on best practices from both the public health and private sector. This model contains a comprehensive approach to increase awareness, screening, treatment and control of hypertension. As multiple similarities have been identified between slum dwellers in Nairobi and African migrants in high-income countries in terms of social circumstances, cultural beliefs and thoughts around hypertension and its management, the SCALE UP model seems a viable model for improving CVD outcomes among African migrant and other vulnerable populations in high-income countries.

## Additional file

Additional file 1:
**Manual for roll out SCALE UP.**

## Abbreviations

CVD: Cardiovascular diseases
LMICs: Low and middle income countries
APHRC: African Population and Health Research Center
AIGHD: Amsterdam Institute for Global Health and Development
SMS: Short message service
