# Supplementary material for: Cardiovascular prevention model from Kenyan slums to migrants in the Netherlands
Source: Global Health. 2015 Mar 7;11:11. doi: 10.1186/s12992-015-0095-y (PMC4363048; doi:10.1186/s12992-015-0095-y)
Supplement: Additional file 1: — Manual for roll out SCALE UP. [file 12992_2015_95_MOESM1_ESM.pdf]

Manual for roll out SCALE UP

Following the manual below allows for making required adjustment to the model for the new targeted location

1 Risk Factors

Determine the relative importance of the different risk factors for CVD in the target location

- In case of different prevalence: adjust focus of the intervention model to relevant root causes

Design of the intervention model

2 Awareness

Determine the best option for the awareness campaign

- Which channels are required for political buy-in?
- Which channels are trustworthy?

3 Access to screening

Detail screening campaign based on local situation

Determine the target age group

- What is the prevalence of hypertension in different age groups?

4 Seeking treatment

Detail incentives for patients based on local situation

5 Long term compliance

Detail incentives for patients based on local situation

Determine the incentive scheme for the community health coordinator (steps 4 and 5)

- Incentive for 'right referrals to the clinic'
- Incentive for long term compliance

6 Determine the overall cost effectiveness of the intervention model

- Using the provided excel tool, an estimation of the overall costs can be made based on the cost assessment per [illegible]
- Using the provided excel tool, an estimation of the health benefits can be made

Step 1: Determine the importance of Risk Factors for CVD in the targeted location

Risk Factors

Intervention model

Awareness | **Access to screening** | **Seeking treatment** | **Long term compliance**

Impact of risk factor on burden of CVD[1] (DALYs) in SSA

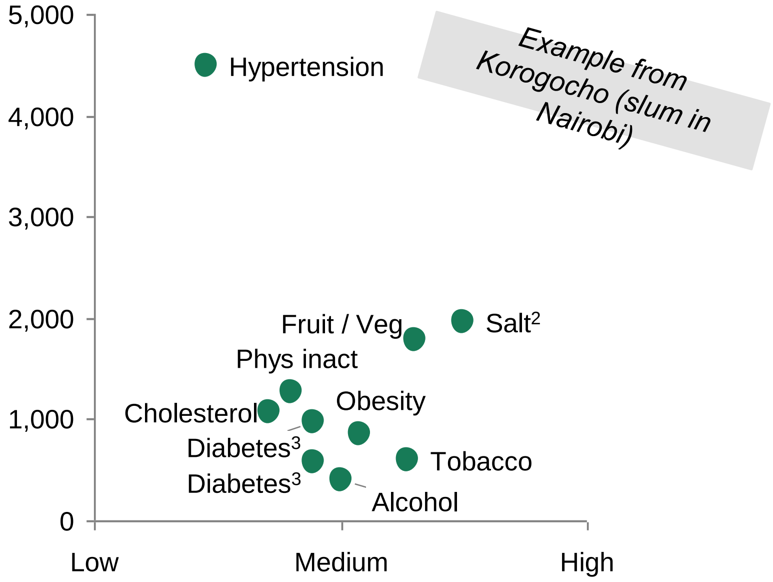

Relative prevalence of risk factor in Nairobi slums compared to African prevalence

Determine the importance of Risk factors for CVD in the target location (x-axis) compared with the importance of risk factors for CVD in SSA (y-axis)

- Hypertension will be the key focus of the intervention
- Dependent on the prevalence of other behavioral risk factors, the counseling sessions will be adjusted in order to incorporate the most important behavioral risk factors

1. Defined as impact on global burden of disease (DALYs)
2. For salt impact is calculated by using method of Strazullo et al, BMJ 2009
3. for Diabetes, impact is calculated based upon total deaths instead of DALYs

Source: WHO: Global Atlas on cardiovascular disease prevention and control, WHO-Global Burden of Disease and Risk Factors- 2006, WHO-Global status report on non communicable diseases 2010; Cochrane review on multiple risk factor interventions for primary prevention of coronary heart disease, 2011 ; CVD study APHRC

Step 2a: Tailor the awareness campaign to the local situation

Risk Factors | Intervention model: **Awareness** → Access to screening → Seeking treatment → Long term compliance

Aim

The aim of the awareness campaign is twofold

1. Create buy-in from the Key Opinion Leaders regarding the screening
- It is fundamental to have the Key Opinion Leaders on board to promote advocacy and to avoid negative communication

2. Create awareness for the screening campaign that will be held by the Community Health Workers (CHW)
- Announcing the screening campaign upfront will likely increase the number of people accepting the BP screening

Requirements

Minimum costs should be spent on the awareness campaign, as a mass screening will be held door-to-door
- No social mobilization required, as people will be screened at their own houses

Sequence of events important: Important to start with the Key Opinion Leaders after which the general population can be informed
- Important to determine who the Key Opinion Leaders in the village are, as they need to be informed first

Detailing in Korogocho

I) Baraaza (Community gathering)
- Key instrument to create buy-in from the Key Opinion Leaders in the village
  - In addition, word of mouth communication is an additional benefit of the Baraaza

II) Religious services
- The various religious services (both churches and mosques) serve as a cheap channel for creating public awareness on the screening campaign
  - CHWs are asked (as part of their job) to inform people about the awareness campaign during various religious services

III) Radio 'jingles'
- Radio jingles on the local radio to create awareness on the upcoming screening campaign

Step 2b: Determine cost effectiveness of different options for the awareness step using this framework

Risk Factors | Intervention model: **Awareness** | Access to screening | Seeking treatment | Long term compliance

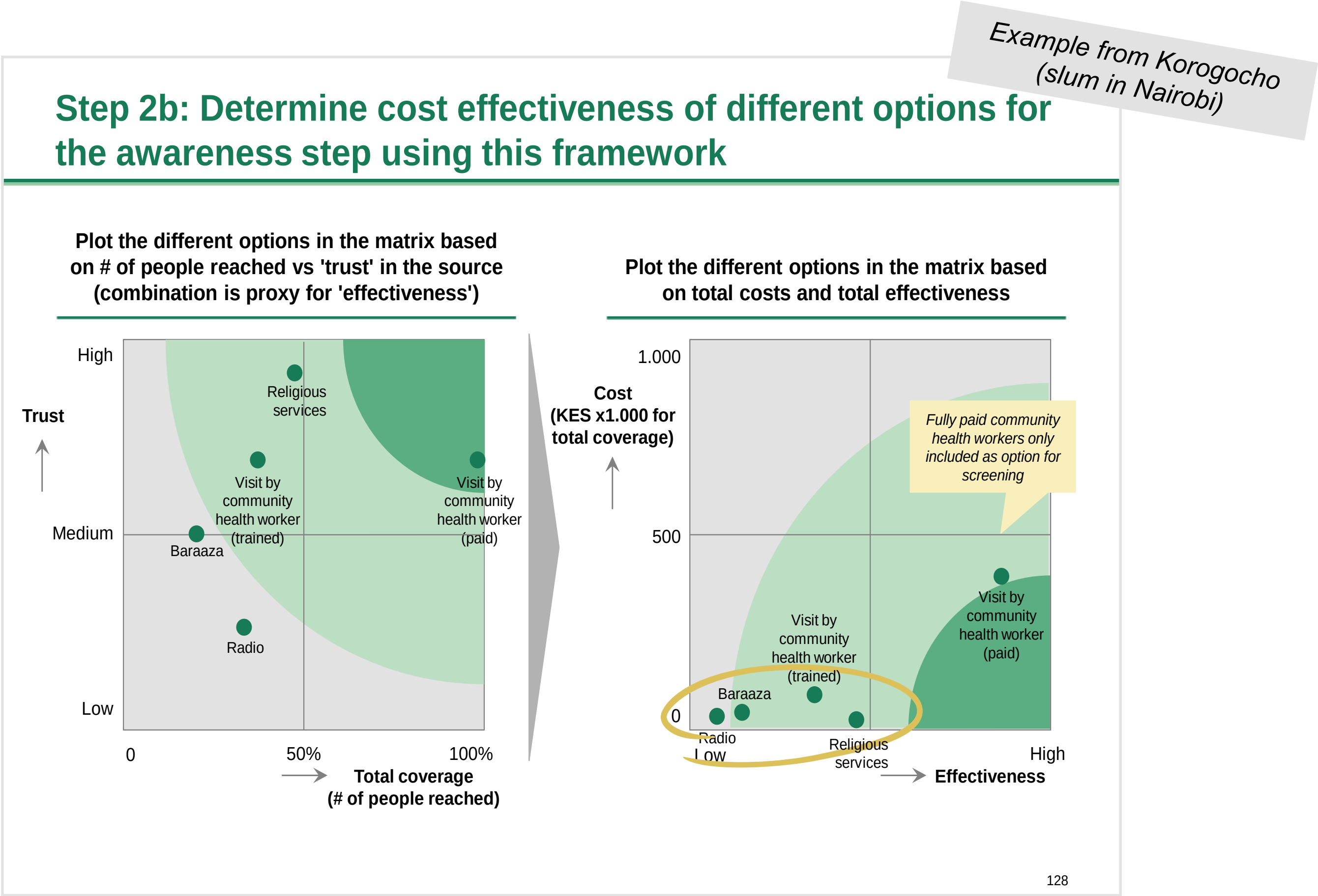

Step 3a: Tailor the door-to-door campaign to the local situation

Risk Factors | Intervention model

Awareness | **Access to screening** | Seeking treatment | Long term compliance

Aim

The door-to-door campaign is a way to maximize the number of people informed about their personal risk on CVD

- Mass screening will be done for Blood Pressure measurements

In addition, counseling on the relationship between a healthy lifestyle and the risk on CVD will be provided to all people screened

- Both people in the medium/high risk group as in the low risk group will receive counseling

Requirements

Use door-to-door screening to lower the efforts required from the people and increase the number of people screened

- People most often are not aware of the risk and should be motivated to get screened
- Door-to-door screening allows for maximum number of people to be screened

Make use of an existing health service mechanism

- To enhance sustainability
- To strengthening the local health service

Determine the target age group for screening

- Based on prevalence of hypertension in different age groups (see next slide)

Detailing in Korogocho

The model in Korogocho used the Community Health Workers to perform door-to-door screening

- CHW are responsible for the health of the people living in their 'unit' of the village
- CHW are often used for health services campaigns

A special incentive scheme has been developed to provide the right motivation for the CHW

- A combination of a fixed and variable fee (see detailed intervention scheme)

The community health workers that provide door-to-door screening enable the sustainability of the model by also creating awareness and motivating patients for long-term compliance

Step 3b: determine the target age group for screening

Goal is to target a small portion of the population, but the majority of hypertensive cases

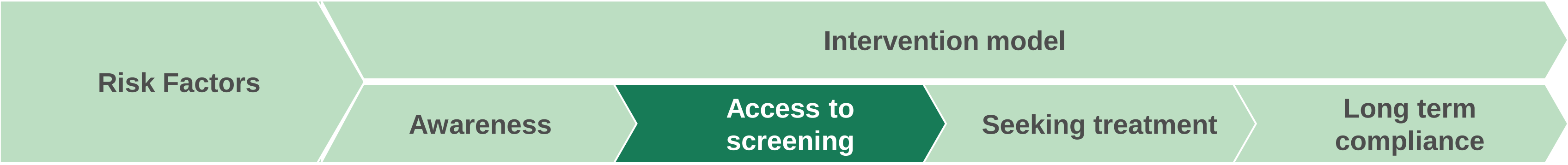

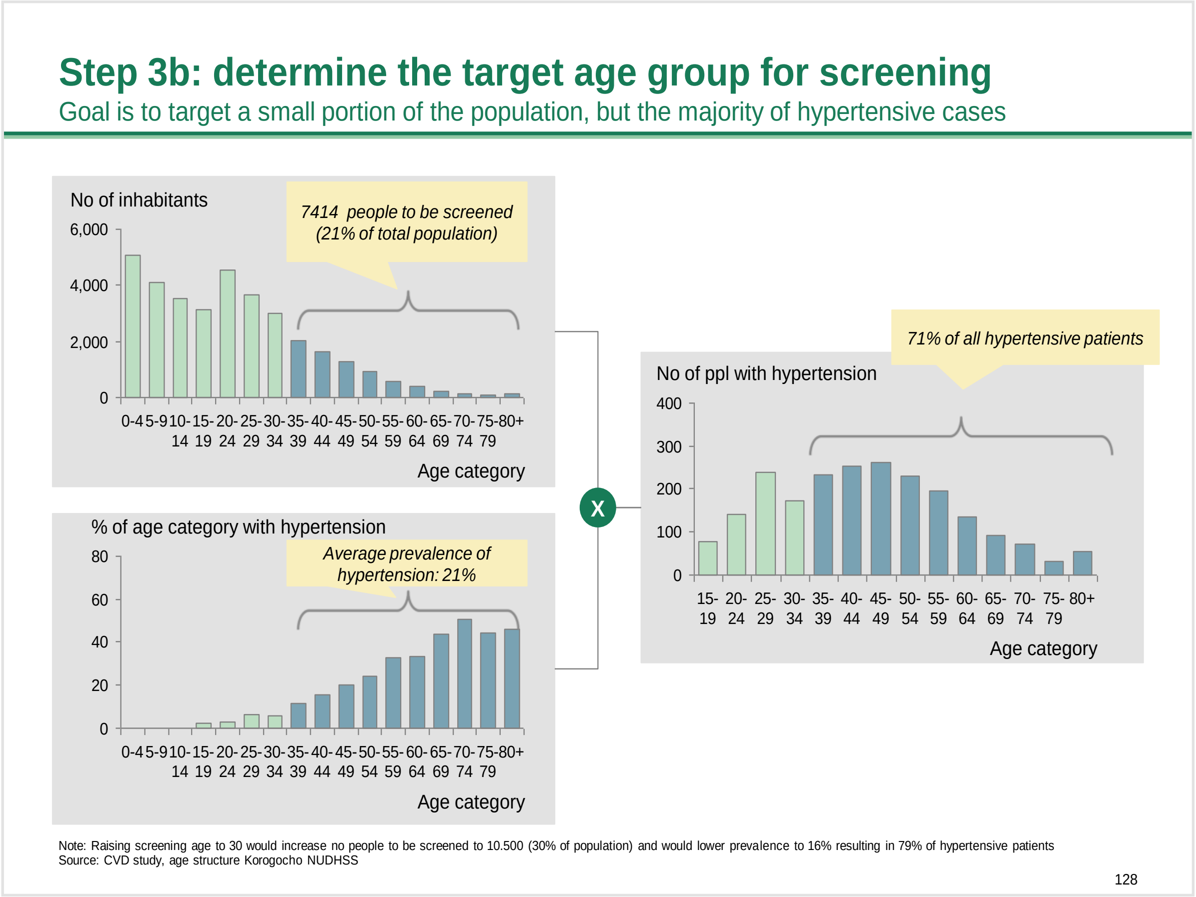

Step 4: Tailor the incentives for 'seeking treatment' to the local situation

Risk Factors | Intervention model: Awareness → Access to screening → **Seeking treatment** → Long term compliance

Aim

From the people that have been diagnosed with medium/high risk, the aim is to maximize the number of people actually going to the (primary care) clinic for treatment

- Through incentives for the patient
- Through incentives for the community health workers

Requirements

The incentive that is offered to people to motivate them to seek treatment, should

- a) be clearly linked to the health service offered
  - i.e. not a gift/present that is not related to the health service provided
- b) should be clearly linked to the real value of the treatment
  - Offering a free treatment should only be done through something that represents the value of that treatment, in order to create awareness of the 'real' costs of treatment

The incentive for the community health worker should motivate the community health worker to make sure that the people with high risk will actually go to the clinic

- Only people that show up at the clinic will result in payment for the

Detailing in Korogocho

In the Korogocho model, the community health worker distributes a voucher to people with medium/high risk. The voucher represents the value of a first treatment

- Mechanism required to validate distribution of vouchers, to avoid misusage
  - For example through validation by team leader of CHWs or by validation of rightfully referred patients in the clinic

Step 5: Tailor the incentives for 'long term compliance' to the local situation

Risk Factors

Intervention model

Awareness | Access to screening | Seeking treatment | **Long term compliance**

Aim

From previous studies it is known that most patients that drop-out of a program, do so within the first six months

For this reason it is critical to make sure patients stay in the program at least during the first six months

Requirements

Interventions for patients to stay long-term compliant should be developed on three levels

- Patient level
  - Social support group / group discount for staying long-term compliant / sms as a reminder for follow-up
- Community health coordinator level
  - Via an incentive scheme for the CHW in case 'his/her' patients stay compliant
- Primary care clinic level
  - Requirement to reduce waiting times to minimize opportunity costs for the patients

Detailing in Korogocho (of the patient interventions)

Patient support group

- To buy directly from a non-for-profit medicine supplier, which reduces the prices of medication significantly
- For social support
- To facilitate 'train the trainer' sessions on relevant topics (for example cooking lessons for a healthier diet)

Reward for long-term compliance

- Measured by compliance of the whole

Weekly sms to the patient

- To remind the patient about medication use and to remind the patient about follow-up visits

Step 6: Determine cost effectiveness of the overall model

Use the excel tool to estimate the overall costs of the model

Example from Korogocho (slum in Nairobi)

	YEAR 1												
					Treatment			Compliance meds					
	PPL starting in model	Awareness	Access to screening	People with high risk	Incentive	Consultation	Meds	Incentive for new patients	Incentive	Consultation	Meds		
	7414	Light & Radio	Door-2-door	People with high r	Free consultation (voucher) + incentive CHW	Consultation	Anti-Hypertensive	Incentive for CHW	Train trainer CBO, SMS, Subsidizing MEDs CBO	Stay in clinic	Anti-Hypertensive		
**Leakage (%)**		85%	90%	21%	80%	100%	100%	100%	65%	100%	100%		
**Variable costs (KES)**		0	28	0	250	208	68	300	104	702	820		
**Fixed costs (KES)**		57,500	430,300	0	0	0	0	307,600	64,500	96,000	48,000		
**Start up costs (KES)**		24,000	1,006,000	0	0	20,000	0	0	0	0	0		
**# of patients enrolled**		7,414	6,302	5,672	1,191	953	953	953	953	619	619		
**Total Costs**		81,500	1,612,753		238,212	218,284	65,146	593,454	163,596	530,601	556,140		
Of which patient costs							*65,146*				*409,044*		
**Program management**												1,200,000	
**TOTAL COSTS**												**5,259,687**	
		0	176,453	0	238,212	198,284	65,146	285,854	99,096	434,601	508,140		

Use the excel tool to estimate the anticipated health impact

Example from Korogocho (slum in Nairobi)

**High risk**				
	0.25	676		
gender	*male*		*female*	
	0.6	405	0.4	270
smoking	*yes*	*no*	*yes*	*no*
	0.2	0.8	0.1	0.9

**Low risk**				
	0.75	3,960		
gender	*male*		*female*	
	0.6	2,376	0.4	1,584
smoking	*yes*	*no*	*yes*	*no*
	0.2	0.8	0.05	0.95

**group**	**1**	**2**	**3**	**4**
N/group	81	324	27	243
**Risk profile**				
gender	male	male	female	female
smoking	yes	no	yes	no
age	47	47	47	47
BP	160	160	160	160
BMI	26	26	26	26

	**5**	**6**	**7**	**8**
	475	1,901	79	1,505
**Risk profile**				
gender	male	male	female	female
smoking	yes	no	yes	no
age	47	47	47	47
BP	120	120	120	120
BMI	24	24	24	24

10y CVD risk	23%	12%	14%	8%		13%	7%	6%	3%	
CVD events predicted	19	39	4	19	80	64	130	5	50	249
